# Ten-Day versus 14-Day Levofloxacin-Containing Triple Therapy for Second-Line Anti-*Helicobacter pylori* Eradication in Taiwan

**DOI:** 10.1155/2013/932478

**Published:** 2013-10-22

**Authors:** Wei-Chen Tai, Chien-Hua Chiu, Chih-Ming Liang, Kuo-Chin Chang, Chung-Mou Kuo, Yi-Chun Chiu, Keng-Liang Wu, Ming-Luen Hu, Yeh-Pin Chou, Shue-Shian Chiou, King-Wah Chiu, Chung-Huang Kuo, Tsung-Hui Hu, Ming-Tsung Lin, Seng-Kee Chuah

**Affiliations:** ^1^Division of Gastroenterology, Department of Internal Medicine, Kaohsiung Chang Gung Memorial Hospital, and Chang Gung University College of Medicine, Kaohsiung 833, Taiwan; ^2^Division of General Medicine, Department of Internal Medicine, Kaohsiung Chang Gung Memorial Hospital, Kaohsiung 833, Taiwan

## Abstract

Second-line *Helicobacter pylori* (*H. pylori*) eradication with fluoroquinolone-containing triple therapy is one of the recommended treatment options, but neither 7-day nor 10-day regimens provide >90% success rates. The current retrospective study aimed to clarify the effects of 10-day and 14-day levofloxacin-containing triple therapies for second-line *H. pylori* eradication in a Taiwanese cohort and to evaluate the potential clinical factors influencing eradication. A total of 200 patients who failed *H. pylori* eradication using the standard triple therapy were prescribed with either a 10-day (EAL-10) or a 14-day (EAL-14) levofloxacin-containing triple therapy group (levofloxacin 500 mg once daily, amoxicillin 1 g twice daily, and esomeprazole 40 mg twice daily). Follow-up studies to assess treatment response were carried out 8 weeks later. Eradication rates attained by EAL-10 and EAL-14 were 75.6%; 95% CI = 63.9–85.3% and 92.5%; 95% CI = 84.5–98.1%, *P* = 0.002 in the per protocol analysis and 68%; 95% CI = 56.6–78.5% and 86%; 95% CI = 76.8–93.4%, *P* = 0.002 in the intention-to-treat analysis. The duration of *H. pylori* therapy is the independent risk factor of *H. pylori* eradication (*P* = 0.003). In conclusion, 14-day levofloxacin-containing triple therapy can provide a >90% *H. pylori* eradication rate, but 10-day treatment duration may be suboptimal. The longer duration of *H. pylori* therapy (14 days) is the independent risk factor.

## 1. Introduction

Many gastrointestinal diseases, either benign or malignant, are associated with *Helicobacter pylori* (*H. pylori*) infections, including peptic ulcer diseases, gastric adenocarcinoma, and gastric mucosa-associated lymphoid tissue lymphoma (MALToma) [1–3]. The successful rate of standard first-line triple therapies using a proton pump inhibitor (PPI), clarithromycin, and either amoxicillin or metronidazole for 7 to 14 days has dropped to less than 80% in many countries especially in areas of high clarithromycin resistance [4–7]. The Maastricht IV/Florence consensus report states that the standard treatment to eradicate *H. pylori *infection is triple therapy; using a proton pump inhibitor (PPI), clarithromycin, and either amoxicillin or metronidazole for 7 to 14 days is recommended for first-line empirical treatment in areas of low clarithromycin resistance, while bismuth-containing quadruple therapy is also an alternative [8]. In areas of high clarithromycin resistance, bismuth-containing quadruple therapies, sequential treatment, or nonbismuth quadruple therapy is used for first-line empirical treatment.

Avoiding problems due to antibiotic resistance has become an important issue when deciding a second-line therapy for *H. pylori* infection [9–11]. Quinolone has the disadvantage of easily acquired drug resistances [3]. It is therefore an important issue to prescribe it wisely targeting at achieving a high eradication rate. A quinolone-containing triple therapy is recommended by both the Maastricht IV/Florence-Consensus Report and the second Asia-Pacific Consensus Guidelines [8, 12]. However, even large meta-analyses of second-line *H. pylori *eradication with fluoroquinolone-based triple therapy have shown that neither 7 days nor 10 days of therapy provide 90% or better treatment success [13]. Previous publications in Taiwan used 7-day levofloxacin-containing therapy and attained 75.3–80.3% of eradication rates [10, 12, 14, 15]. However, the reports on second-line eradication by using 14-day levofloxacin-containing triple therapy are few in the literature.

The current retrospective study aimed to clarify the effects of 10-day (EAL-10) and 14-day (EAL-14) esomeprazole/amoxicillin/levofloxacin therapy for patients who failed to have *H. pylori *eradicated after standard triple therapy in Taiwan and to determine the potential clinical factors influencing the eradication.

## 2. Materials and Methods

### 2.1. Patients

A total of 200 *H. pylori*-infected patients who failed *H. pylori* eradication using the standard triple therapy (PPI twice daily, 500 mg of clarithromycin twice daily, and 1 g of amoxicillin twice daily) for 7 days were recruited. All the patients were at least 18 years of age and had received endoscopic exam which showed peptic ulcers disease or gastritis. The confirmations of *H. pylori* eradication failure were defined as positive results for both the rapid urease test and histology after first-line eradication. The criteria for exclusion were (a) ingestion of antibiotics, bismuth, or PPI within 4 weeks, (b) allergic history to the medications used, (c) previous gastric surgery, (d) the coexistence of serious concomitant illness (e.g., decompensated liver cirrhosis and uremia), and (e) pregnancy. These 200 patients were prescribed with either a 10-day levofloxacin-containing triple therapy group (levofloxacin 500 mg once daily, amoxicillin 1 g twice daily, and esomeprazole 40 mg twice daily for 10 days, EAL-10) or a 14-day levofloxacin-containing triple therapy group (EAL-14). Patients were followed up to assess the adverse effects and drug compliance after they finished the medications. All patients received either an endoscopy or a urea breath test eight weeks later. Besides, we also performed a backup urea breath test on all participants to avoid any false-negative results. The definition of poor compliance was that the patient failed to finish 80% of all medications due to adverse effects [5, 15].

This study was approved by both the Institutional Review Board and the Ethics Committee of Chang Gung Memorial Hospital (IRB102-0921B). All patients provided their written informed consent before endoscopic interventions.

### 2.2. Outcomes

The primary endpoint was the successful eradication of *H. pylori*. There was additional analysis of adverse events during therapy.

### 2.3. Diagnosis of *Helicobacter pylori* Infection

#### 2.3.1. Rapid Urease Test

The rapid urease test involved the collection of gastric antrum biopsy specimens by endoscopy, which were tested using a urea agar base enriched with 40% urea solution (eUAB, Oxoid) and a commercial rapid urease test (Pronto Dry, Medical Instrument Corp, Switzerland) [16]. The results of the rapid urease test were interpreted as positive if the color of the gel turned pink or red when examined after 1 h at room temperature.

#### 2.3.2. Urea Breath Test

The urea breath test was performed according to our previous studies [17]. The cut-off value was set at 4.8% of *δ* ^13^ CO_2_. Staffs were blinded to the *Helicobacter pylori* status performed to the test.

### 2.4. Statistical Analysis

The primary outcome variables were the rates of eradication, the presence of adverse events, and the level of patient compliance. Using the SPSS program (Statistical Package for the Social Sciences version 18, Chicago, IL, USA), the chi-square test with or without Yates's correction for continuity and Fisher's exact test were used to compare the major outcomes between groups. Eradication rates were analyzed by both the intention-to-treat (ITT) and per protocol (PP) approach. ITT analysis included all assigned patients who had taken at least one dose of the study medication. Patients whose infection status was unknown following treatment were considered treatment failures for the purposes of the ITT analysis. The PP analysis excluded patients with unknown *H. pylori* status following therapy and those with major protocol violations. A *P* value of less than 0.05 was considered statistically significant. To determine the independent factors that affected the treatment response, clinical parameters were analyzed by univariate and multivariate analyses.

## 3. Results

**Figure 1:**
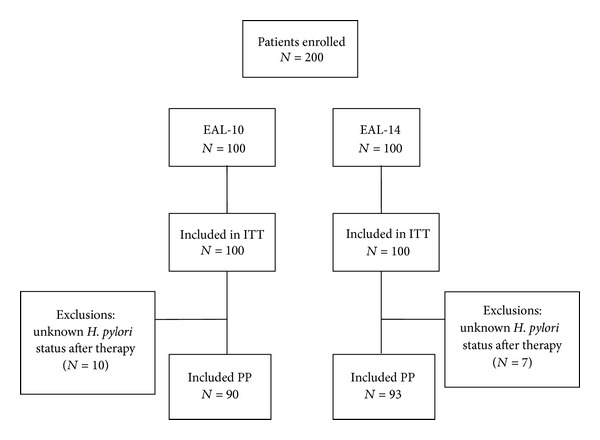
Disposition of patients.

A total of 200 patients were enrolled (100 each in the EAL-10 and the EAL-14 group). Ten patients lost to follow-up in EAL-10 group and 7 in the EAL-14 group resulted in 90 in the PP study for EAL-10 and 93 for EAL-14 (Figure 1). The demographic data of the two groups are summarized in Table 1, and none of the variables showed significant difference between EAL-10 and EAL-14 groups.

Eradication rates attained by EAL-10 and EAL-14 were 75.6%; 95% CI = 63.9–85.3% and 92.5%; 95% CI = 84.5–98.1%, *P* = 0.002 in the PP analysis and 68%; 95% CI = 56.6–78.5% and 86%; 95% CI = 76.8–93.4%, *P* = 0.002 in the ITT analysis (Table 2).

### 3.1. Adverse Events and Complications

The adverse events were 11% (11/100) in EAL-10 group and 16% (16/100) in EAL-14 group (Table 2). These adverse events were abdominal pain, constipation, diarrhea, dizziness, headache, nausea/vomiting, and skin rash, but they were mild and had little disturbance in patients' daily activities (Table 3). Both groups had good drug compliances (100% in EAL-10 group versus 99% in EAL-14 group).

### 3.2. Factors Influencing the Efficacy of the Anti-*H. pylori* Therapies

Univariate analysis showed that the duration of *H. pylori *eradication (*P* = 0.002) was the clinical factor influencing the efficacy of *H. pylori *eradication therapy (Table 4). Simultaneously, multivariate analysis showed the duration of *H. pylori* eradication (EAL-10 versus EAL-14, OR: 3.98, 95% CI: 1.60–9.84, *P* = 0.003) was the independent risk factor of successful *H. pylori *eradication (Table 5).

## 4. Discussion

Quinolone-containing triple therapy is one of the recommended second-line therapies after the failure of the standard first-line empirical clarithromycin-containing therapy, with bismuth-containing quadruple therapy as an alternate [8]. However, we are also aware that bismuth salts are not available in many hospitals. As a matter of fact, such triple therapy with quinolone-containing regimens has been shown to be a good alternative treatment as a second-line *H. pylori *therapy with comparable results to the recommended bismuth-based quadruple therapy [18–20]. Large meta-analyses of second-line *H. pylori *eradication with fluoroquinolone triple therapy have shown that 7 to 10 days of therapy could not provide 90% or better treatment success [14, 21]. Published papers on the efficacies of 14-day quinolone-containing triple therapy for second-line therapy are very few, and none of them offered head-to-head data on efficacies for EAL-10 and EAL-14 therapies in similar cohort. This is a very important message because one must target to eradicate the bacteria with a better formula to avoid subsequent quinolone resistance if the eradication failed.

Levofloxacin is a levorotatory isomer of ofloxacin with known activity against many Gram-negative and Gram-positive bacteria [22]. The mode of action of levofloxacin is based on the inhibition of bacterial DNA topoisomerase II. A levofloxacin-containing triple therapy is simple and well tolerated and has high compliance (100% and 99% in the current study). The relatively low incidence of adverse events among the EAL group was the key factor related to this good compliance. This is important because compliance plays a cardinal role in eradication. In addition, *in vitro *levofloxacin retains its activity even in dual *H. pylori *resistant strains to clarithromycin and metronidazole [23, 24]. Similar effects have been observed *in vivo*, showing that most of the dual metronidazole and clarithromycin resistances in *H. pylori* infections are cured with the levofloxacin-containing regimen [18, 25]. Moreover, there is an *in vivo* synergistic effect of the quinolone antimicrobial agent and the proton-pump inhibitor on strains of *H. pylori* [26].

Drug resistance to antibiotics is an important key factor in successful *H. pylori* eradication. Interestingly, it was just about a decade ago that levofloxacin was chosen as the most promising agent used to overcome antimicrobial resistance among the new antibiotic and drug combinations that had been evaluated, including fluoroquinolones, rifabutin, furazolidone, and azithromycin [27]. Gisbert et al. reported that levofloxacin triple scheme was superior to quadruple therapy (81% versus 70%) with a lower incidence of side effects (19% versus 44%). Again, the 10-day levofloxacin-based triple scheme was superior to the same 7-day therapy (81% versus 73%) [13]. Today, quinolone resistance is becoming a major concern for the EAL therapy. Just like metronidazole and clarithromycin, drug resistance to levofloxacin is becoming an important factor responsible for unfavorable results. In Taiwan, Kuo et al. reported that levofloxacin-resistant strain was found in 28.3% of patients [11]. In fact, primary levofloxacin resistance has been increasing in most parts of the world with values of 5.5% to 32.3% in countries such as Japan, Brazil, Italy, Hong Kong, and Republic of Korea [28–32]. Therefore, it is very important that the use of quinolone-containing triple therapies need cautious monitoring, because Taiwan is an endemic area for tuberculosis infection [33].

Another reason for the failure of quinolone-containing triple therapies as second-line eradication regimens is the duration of the treatment instead of the dosage. Both the univariate and multivariate analyses in the current study showed that the length of *H. pylori *treatment was the clinical factor influencing the efficacy of eradication. This was similar to Caro and colleagues' report that the duration of treatment is the crucial factor influencing eradication rate but not dosage [20]. In the systemic review reported by Gisbert et al, higher *H. pylori *cure rates with a 10-day rather than a 7-day regimen were found with the levofloxacin-amoxicillin-PPI combination (80% versus 68%), suggesting that the longer (10-day) therapeutic scheme should be chosen for levofloxacin-containing triple therapy [13]. In Taiwan, Cheng et al. also reported that prescribing 500 mg and 1000 mg levofloxacin per day did not affect the eradication rates [14]. Liou et al. also attained only 76.9% eradication with levofloxacin 750 mg once daily [34]. In both studies, the length of treatment was only 7 days. The current study showed that EAL-10 could be suboptimal and only attained 75.6% eradication rate in the PP analysis. None of these studies with 7–10 days of levofloxacin-containing triple therapies attained 90% or better treatment success. Two recently published studies reported that extending the length of quinolone-containing triple therapies to 14 days could achieve eradication success up to 95% (moxifloxacin) and 93.6% (levofloxacin) [35, 36]. Consequently, the current study achieved an eradication rate of 92.5% in the EAL-14 group in PP analysis but only 75.6% in the EAL-10 group. Our study result adds on a potentially important message that 14 days should be the optimal length of treatment for quinolone-containing triple therapies as a second-line *H. pylori* treatment option instead of the 7–10-day regimens. The bottom line is that quinolone resistance is carefully monitored.

However, the current study encountered its limitations. First, since our laboratory could not perform CYP2C19 genotyping, we used an esomeprazole-based regimen because it had minimal first-pass metabolism and had a greater gastric acid suppression effect than omeprazole. Second, there was the lack of information regarding the prevalence of antimicrobial resistance.

## 5. Conclusions

A 14-day levofloxacin-containing triple therapy can provide a >90% *H. Pylori* eradication rate, but 10-day treatment duration may be suboptimal. The longer duration is the independent risk factor for eradication. This is a very important message since quinolone easily acquires resistance. Meanwhile, a continuous search for novel second-line therapeutic approaches which are cost-effective and minimize drug resistance to cure *H. pylori* infection is still mandatory.

## Figures and Tables

**Table 1:** Demographic data and endoscopic appearances of the two patient groups.

	EAL-10 (*n* = 100)	EAL-14 (*n* = 100)
Characteristics		
Age (year) (mean ± SD)	55.6 ± 13.2	57.6 ± 12.8
Gender (male/female)	44/56	45/55
Smoking	9 (9%)	6 (6%)
Alcohol consumption	8 (8%)	13 (13%)
Previous history of peptic ulcer	21 (21%)	29 (29%)
Endoscopic findings		
Gastric ulcer	36 (36%)	35 (35%)
Duodenal ulcer	18 (18%)	20 (20%)
Gastric and duodenal ulcer	11 (11%)	14 (14%)
Unspecified (include gastritis)	35 (35%)	31 (31%)

**Table 2:** The major outcomes of EAL-10 and EAL-14 eradication therapy.

	Eradication rate		
	EAL-10 (*n* = 100)	EAL-14 (*n* = 100)	*P* value
Intention-to-treat	68% (68/100)	86% (86/100)	0.002
Per-protocol	75.6% (68/90)	92.5% (86/93)	0.002
Adverse event	11% (11/100)	16% (16/100)	0.301
Compliance	100% (100/100)	99% (99/100)	1.000

EAL-10: esomeprazole/amoxicillin/levofloxacin triple therapy × 10 days and EAL-14: esomeprazole/amoxicillin/levofloxacin triple therapy × 14 days.

**Table 3:** Adverse events during EAL-10 and EAL-14 therapies.

Adverse events	EAL-10 (*n* = 100)	EAL-14 (*n* = 100)	*P* value
Abdominal pain	5	7	0.552
Constipation	2	2	1.000
Diarrhoea	0	4	0.121
Dizziness	4	1	0.369
Headache	6	3	0.498
Nausea/vomiting	2	2	1.000
Skin rash	0	2	0.497

EAL-10: esomeprazole/amoxicillin/levofloxacin triple therapy × 10 days and EAL-14: esomeprazole/amoxicillin/levofloxacin triple therapy × 14 days.

**Table 4:** Univariate analysis of the clinical factors influencing the efficacy of *Helicobacter  pylori* eradication.

Principle parameter	Case no.	Eradication rate	*P* value
Age			
<60 years	111	82.0% (91)	0.318
≥60 years	72	87.5% (63)	
Sex			
Female	98	81.6% (80)	0.316
Male	85	87.1% (74)	
Smoking			
(−)	168	83.3% (140)	0.472
(+)	15	93.3% (14)	
Previous history of peptic ulcer			
(−)	138	81.9% (113)	0.141
(+)	45	91.1% (41)	
HP eradication (per protocol)			
EAL-10	90	75.6% (68)	0.002
EAL-14	93	92.5% (86)	
Compliance			
Good	183	84.2% (154)	—
Poor	0	0% (0)	

EAL-10: esomeprazole/amoxicillin/levofloxacin triple therapy × 10 days and EAL-14: esomeprazole/amoxicillin/levofloxacin triple therapy × 14 days.

**Table 5:** Multivariate analysis of the clinical factors influencing the efficacy of *Helicobacter pylori *eradication.

Clinical factor	Coefficient	Standard error	Odds ratio (95% CI)	*P* value
Duration of *H. pylori* eradication (10 days versus 14 days)	1.38	0.46	3.98 (1.60–9.86)	0.003

EAL-10: esomeprazole/amoxicillin/levofloxacin triple therapy × 10 days and EAL-14: esomeprazole/amoxicillin/levofloxacin triple therapy × 14 days.
